# Fabrication of Magnetically and Photothermally Functionalized Materials Based on Corn Stalk Pith Framework for Oil–Water Separation

**DOI:** 10.3390/polym18070860

**Published:** 2026-03-31

**Authors:** Yutong Cui, Xin Shu, Boyu Cui, Jiayan Ding, Wei Dai, Chunmao Yang, Weihong Wang

**Affiliations:** Engineering Research Center of Advanced Wooden Materials, Ministry of Education, Northeast Forestry University, Harbin 150040, China; yutongcui1996@gmail.com (Y.C.); shuxin09181323@163.com (X.S.); boyucui0124@163.com (B.C.); wydingjiayan@163.com (J.D.); weiddddv@163.com (W.D.); young112023@163.com (C.Y.)

**Keywords:** biomass-derived three-dimensional porous sorbent, superhydrophobic oilwater separation, photothermal conversion, in situ viscosity reduction in heavy crude oil, magnetic-responsive recovery

## Abstract

To address critical challenges in marine oil spill remediation, including limited penetration of high-viscosity crude oil and inefficient adsorbent recovery, it is imperative to develop environmentally friendly materials integrating high-efficiency adsorption, in situ viscosity reduction, and controllable recovery. In this study, a delignified corn pith (CPDL) with a three-dimensional porous structure was employed as a green matrix. Through constructing a Fe_3_O_4_/expansible graphite (EG)/polyvinylidene fluoride (PVDF) composite functional coating combined with silanization modification, a multifunctional biomass-based oil sorbent (Fe_3_O_4_/EG/PVDF-CPDL) was successfully fabricated. The material maintains the inherent porous architecture while forming a stable micro/nano-rough surface, exhibiting excellent superhydrophobicity with a water contact angle of approximately 155°, and demonstrating exceptional stability in harsh acidic/alkaline/saline environments and multiple cycles. Benefiting from the synergistic photothermal effect of Fe_3_O_4_ and EG, under one sun illumination (1 kW/m^2^), the material surface temperature rapidly reaches above 80 °C within 100 s, reducing the viscosity of high-viscosity crude oil by over 95% (from 1.39 × 10^5^ to approximately 6.0 × 10^3^ mPa·s), thereby enabling rapid penetration and adsorption within 50 s. Moreover, the composite coating significantly enhances mechanical performance, achieving a compressive strength of 320 kPa (approximately eight times higher than that of the pristine substrate), ensuring structural integrity during handling and compression recovery. Meanwhile, the material demonstrates precise directional manipulation and efficient recovery through external magnetic fields due to its superior magnetic responsivity. Experimental results reveal a broad-spectrum adsorption capacity (14.8–30.2 g/g) and separation efficiency exceeding 96% after 20 adsorption–desorption cycles. In summary, this work presents an innovative strategy with significant application potential for efficient and controllable remediation of marine oil spills, particularly high-viscosity crude oil, by integrating synergistic functions of porous adsorption, superhydrophobic corrosion resistance, photothermal viscosity reduction, mechanical reinforcement, and magnetic control.

## 1. Introduction

Marine oil spill accidents, particularly those involving the release of high-viscosity, low-fluidity crude oil, can form persistent oil films on the seawater surface, thereby markedly retarding the wetting, penetration, and pore transport of the oil phase into porous sorbent materials and consequently compromising the effectiveness of conventional adsorption-based remediation [1]. Unlike low-viscosity oils, high-viscosity crude oil generally exhibits stronger cohesive interactions and poorer fluidity at ambient temperature, making it difficult to rapidly enter the interconnected pores within materials [2]. Meanwhile, the recovery of oil-laden sorbents still commonly relies on manual or mechanical collection, which is not only operationally inefficient but also increases the risk of secondary contamination caused by residual sorbents [3,4]. Therefore, the development of material systems that integrate rapid adsorption, active viscosity reduction, and controllable recovery has become a critical scientific and engineering issue in the design of oil spill remediation materials for high-viscosity marine crude oil spills [5,6].

In recent years, extensive efforts have been devoted to the construction of superhydrophobic/superoleophilic interfaces, solar-driven photothermal conversion, and three-dimensional porous frameworks [7,8,9,10]. The in situ heating of crude oil at the adsorption interface by photothermal materials to reduce viscosity and promote wetting has been regarded as a promising route to overcoming the mass-transfer limitations associated with high-viscosity oils [11,12,13,14,15,16]. Existing studies have typically loaded carbon nanotubes, reduced graphene oxide, Fe_3_O_4_, polydopamine-derived layers, or other photothermal components onto polyurethane sponges, melamine foams, fabrics, or aerogel substrates in order to integrate photothermal responsiveness with selective oil–water separation [17,18,19,20,21]. For example, Si et al. [22] employed graphene as a photothermal agent and utilized a superelastic, compressible chitosan (CS)/Chlorella pyrenoidosa matrix to fabricate a biomass aerogel, which exhibited rapid heating and the capability to remove high-viscosity crude oil under illumination. Wang et al. [23] reported a sandwich-structured photothermally active superhydrophobic composite material, in which crude oil separation and recovery performance were enhanced through multilayer functional synergy. These studies have demonstrated the considerable potential of photothermal-assisted adsorption for the remediation of high-viscosity oils.

However, several issues related to mechanistic synergy and engineering applicability remain to be further addressed in current material systems. First, although interfacial photothermal heating itself has been proven effective in reducing the local viscosity of high-viscosity crude oil, the performance of three-dimensional porous sorbent materials depends not only on whether interfacial viscosity reduction occurs, but also on whether this effect can be further translated into continuous penetration and rapid transport along interconnected pore channels, thereby truly improving the overall adsorption efficiency [24,25,26]. Second, while introducing rough structures, low-surface-energy components, or photothermal particles, certain functional coatings may also affect the intrinsic open-pore architecture and mass-transfer pathways of the substrate, resulting in a trade-off between surface functionalization and pore accessibility; this issue is particularly critical for high-viscosity crude oil with restricted flowability [27,28]. Third, many existing studies have mainly focused on the adsorption or separation process itself, whereas relatively insufficient attention has been paid to low-disturbance post-adsorption recovery, especially magnetically assisted directional manipulation and rapid collection, thus limiting their operational applicability in complex marine environments [29,30]. At the same time, although conventional petroleum-based foam or sponge substrates are easy to process and functionalize, increasing concern has been raised regarding their environmental persistence and the potential risk of microplastic release. In contrast, agricultural waste biomass, which is abundant, renewable, and endowed with an intrinsically porous structure, offers a more attractive alternative for constructing sustainable sorbent scaffolds. Nevertheless, studies on the synergistic integration of photothermal responsiveness, interfacial stabilization, wettability regulation, and magnetically controlled recovery on biomass-based scaffolds remain limited.

Based on the above considerations, a synergistic material design strategy for the remediation of high-viscosity marine crude oil was proposed in this study by targeting the continuous process encompassing “viscosity reduction–wetting–pore transport–adsorption–recovery”. Delignified corn pith (CPDL) was employed as a green three-dimensional porous scaffold, and a Fe_3_O_4_/expanded graphite (EG)/PVDF composite functional coating was constructed on its surface in combination with methyltrimethoxysilane modification to fabricate a multifunctional Fe_3_O_4_/EG/PVDF-CPDL composite oil sorbent. To experimentally validate the proposed continuous mechanism, a systematic characterization strategy was tailored for each step of the process: The viscosity reduction process was verified via photothermal heating tests under simulated solar irradiation (1 kW/m^2^), in which the evolution of surface temperature was monitored using infrared thermal imaging, and the viscosity variation in crude oil at different temperatures was quantitatively measured. The wetting behavior was characterized via water contact angle (WCA) measurements and oil droplet spreading tests, confirming the superhydrophobic/superoleophilic properties of the material surface. The pore transport behavior was validated via scanning electron microscopy (SEM) imaging of the cross-sectional structure, which revealed the well-interconnected channel network within the material. The adsorption capacity and selectivity were quantitatively determined for a wide range of organic solvents and oils via the gravimetric method, and the separation efficiency was evaluated over multiple adsorption–desorption cycles. The magnetic recovery capability of the material was demonstrated via magnetically actuated experiments, where the directional movement and collection efficiency of the material guided by an external magnetic field were visually recorded. Taken together, these experiments establish a complete chain of evidence that fully supports the proposed viscosity reduction–wetting–pore transport–adsorption–recovery sequential mechanism.

In this system, CPDL retained its natural characteristics of light weight, interconnected structure, and high porosity, thereby providing low-resistance pathways for oil entry; EG, as a broadband light-absorbing and highly thermally conductive component, contributed to enhanced solar energy harvesting and heat diffusion; Fe_3_O_4_ not only participated in photothermal conversion but also endowed the material with magnetic responsiveness to external fields; PVDF enhanced the interfacial adhesion between the functional coating and the biomass scaffold as well as the overall mechanical stability; and silanization further reduced the surface energy to construct a stable superhydrophobic interface. As a result, the material system functioned not merely as a passive adsorption carrier, but as an integrated platform capable of actively reducing the viscosity of high-viscosity crude oil under solar irradiation, promoting its rapid penetration into the open pore channels, and enabling controllable post-adsorption recovery under a magnetic field. The essence of this design lies not in the simple superposition of “porous”, “superhydrophobic”, “photothermal”, and “magnetic” attributes, but in the establishment of a closed-loop mechanism suitable for the treatment of high-viscosity oils through the synergistic regulation of material composition and interfacial structure. Specifically, the photothermal components rapidly generated heat to weaken the viscous resistance of crude oil, the superhydrophobic/superoleophilic interface promoted selective wetting, the interconnected pores accelerated bulk oil transport, the reinforced scaffold maintained structural integrity during compression, and the magnetic responsiveness provided an additional means for directional recovery under complex sea surface operating conditions. Therefore, this system simultaneously addresses three key challenges associated with high-viscosity crude oil, namely difficult entry, slow adsorption, and difficult recovery. This work provides a feasible route for constructing oil spill remediation materials that integrate in situ photothermal viscosity reduction, superhydrophobic selective adsorption, mechanical reinforcement, and magnetically controlled recovery on agricultural waste biomass scaffolds, and offers a new material design concept for the green and efficient treatment of high-viscosity marine crude oil.

## 2. Materials and Methods

### 2.1. Materials

Corn stalk (length: 1.5–2 m) was sourced from Xiangfang District, Harbin City, Heilongjiang Province. Sodium chlorite (NaClO_2_, 80% purity) and glacial acetic acid (CH_3_COOH, 98% purity) were purchased from Aladdin Bio-Chem Technology Co., Ltd. (Shanghai, China). Polyvinylidene fluoride (PVDF), dimethylformamide (DMF, 99.5%), nano iron oxide (Fe_3_O_4_, 98% purity), expanded graphite (EP, 99% purity), and methyltrimethoxysilane (MTMS, 98% purity) were obtained from Macklin Biochemical Co., Ltd. (Shanghai, China).

### 2.2. Experimental Method

Corn stalk was subjected to peeling and separation of rind and pith, followed by cutting the pith segments into 2 cm fragments. The cut pith fragments were immersed in 2% (*w*/*v*) NaClO_2_ solution, adjusted to pH 4.6 with glacial acetic acid, and treated at 80 °C for 5 h. After treatment, the samples were washed with deionized water until the filtrate became neutral, then frozen at −18 °C for 12 h and freeze-dried for 24 h to obtain delignified corn pith (CPDL). Fe_3_O_4_ nanoparticles and expanded graphite (EG) powders were dried to constant weight in a vacuum oven at 103 °C. Ten grams of polyvinylidene fluoride (PVDF) were dissolved in 90 mL dimethylformamide (DMF) under magnetic stirring at 60 °C until a homogeneous transparent viscous solution formed. Pre-dried Fe_3_O_4_ (4 g) and EG (4 g) were added to the PVDF solution, followed by mechanical stirring at room temperature for 1 h to prepare a magneto-photothermal hybrid slurry containing Fe_3_O_4_, EG, and PVDF. The mass ratio of Fe_3_O_4_:EG:PVDF in the slurry was 4:4:10. This specific ratio was designed to achieve synergistic functionality. The amount of PVDF (10 g) was selected to provide sufficient viscosity and binding capacity, ensuring the formation of a continuous, robust composite coating that firmly adhering to the porous CPDL scaffold without clogging its intrinsic pores. The equal mass ratio between Fe_3_O_4_ (4 g) and EG (4 g) was chosen to balance their complementary roles: Fe_3_O_4_ nanoparticles serve as an efficient photothermal agent and provide magnetic responsiveness, while EG acts as a broadband light absorber and thermal conductor to enhance and homogenize photothermal conversion efficiency. This composition (Fe_3_O_4_:EG:PVDF = 4:4:10) was optimized through preliminary experiments to ensure the best overall performance in terms of photothermal conversion, magnetic actuation, mechanical stability, and preservation of the substrate’s porous structure for oil transport.

**Figure 1:**
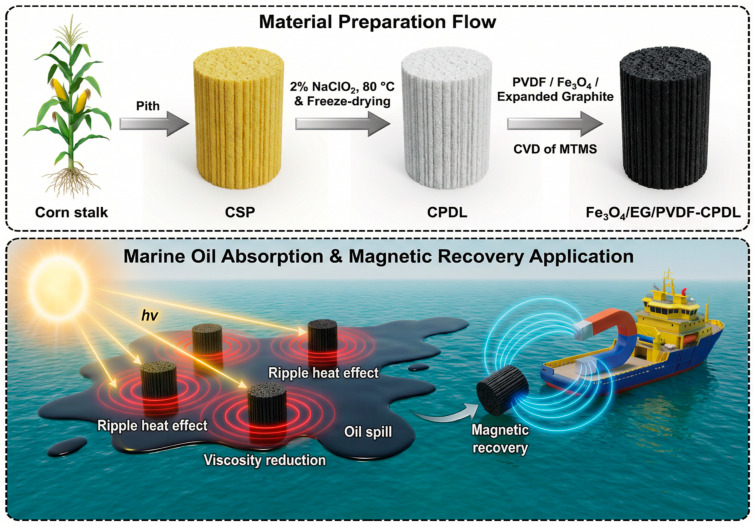
Schematic illustration of the preparation method and application of Fe_3_O_4_/EG/PVDF-CPDL.

The hybrid slurry was uniformly coated onto CPDL samples via brush coating until complete surface coverage and pore infiltration were verified visually. After drying at 60 °C for 4 h, gravimetric measurements revealed a consistent composite coating mass ranging from 95% to 110% of the original CPDL substrate weight (average loading ratio ≈ 1:1) across multiple batches. This loading range ensured sufficient functional material deposition while preserving the structural integrity required for oil transport. Subsequently, the dried samples were placed in a desiccator with a small beaker containing 3 mL MTMS liquid at the bottom, positioned on an upper shelf, and sealed for silanization deposition at 60 °C for 4 h. After removal, the samples were stored in a fume hood at room temperature for 24 h to eliminate unreacted MTMS monomers, yielding the hydrophobically modified magneto-photothermal oil–water separation material designated as Fe_3_O_4_/EG/PVDF-CPDL (Figure 1).

### 2.3. Material Characterisation

The microstructure of the samples was examined using a field-emission scanning electron microscope (SEM, SU-8010, Hitachi, Tokyo, Japan). Prior to analysis, samples were sputter-coated with gold, and an accelerating voltage of 15 kV was applied. Chemical structure was analyzed using a Fourier-transform infrared spectrometer (FTIR, Nicolet iS50, Thermo Fisher, Wilmington, DE, USA); samples were homogenized with KBr powder, pressed into pellets, and scanned over the range of 4000–400 cm^−1^. Crystal structure was characterized by X-ray diffraction (XRD, D8 ADVANCE. Bruker, Karlsruhe, Germany) employing Cu Kα radiation, with a 2θ scanning range of 5–50°. Surface elemental composition and chemical states were determined using an X-ray photoelectron spectrometer (XPS, ESCALAB 250Xi, Thermo Fisher, Wilmington, DE, USA) equipped with an Al Kα radiation source, following Ar^+^ sputter cleaning of the surface prior to measurement. Hydrophobicity was evaluated using a contact angle goniometer (OCA20, Dataphysics, Stuttgart, Germany) by measuring the static water contact angle of a 5 μL deionized water droplet at 25 °C; the reported value represents the mean of five replicate measurements. Oil adsorption capacity was assessed gravimetrically: dried samples were immersed in organic solvents (e.g., n-hexane, dichloromethane) until adsorption equilibrium was attained, followed by weighing. The adsorption capacity (qₑ) was calculated using the equation qₑ = (m_1_ − m_0_)/m_0_, where m_0_ and m_1_ denote the sample masses before and after adsorption, respectively. Photothermal performance was evaluated under AM 1.5 G simulated solar irradiation (1000 W/m^2^); surface temperature evolution of the samples in air and water was recorded using a thermal imaging camera (FLIR T540, Wilsonville, OR, USA). Compressive strength was measured in accordance with ASTM D695 using a universal testing machine at a crosshead speed of 0.5 mm/min until specimen failure. Collectively, these multi-scale characterization techniques provide a comprehensive evidence chain confirming the successful fabrication of the Fe_3_O_4_/EG/PVDF-CPDL composite. Specifically, SEM imaging visually verified the structural integrity of the corn pith framework and the uniform deposition of the hybrid coating within the interconnected pore network. Complementing the morphological analysis, FTIR and XRD patterns confirmed the co-existence of characteristic functional groups and crystalline phases corresponding to Fe_3_O_4_ nanoparticles, expanded graphite, and the PVDF binder, indicating that the components were successfully incorporated without altering their intrinsic chemical identities. Furthermore, XPS analysis offered deeper insights into the surface elemental composition and chemical states, validating the presence of key elements (Fe, F, C, O) and the formation of stable interfacial interactions between the coating and the substrate. Together, these results corroborate that the proposed synthesis strategy effectively constructed a hierarchically structured material with the intended magnetic, photothermal, and hydrophobic functionalities.

## 3. Results

### 3.1. Microscopic Morphology Analysis

**Figure 2:**
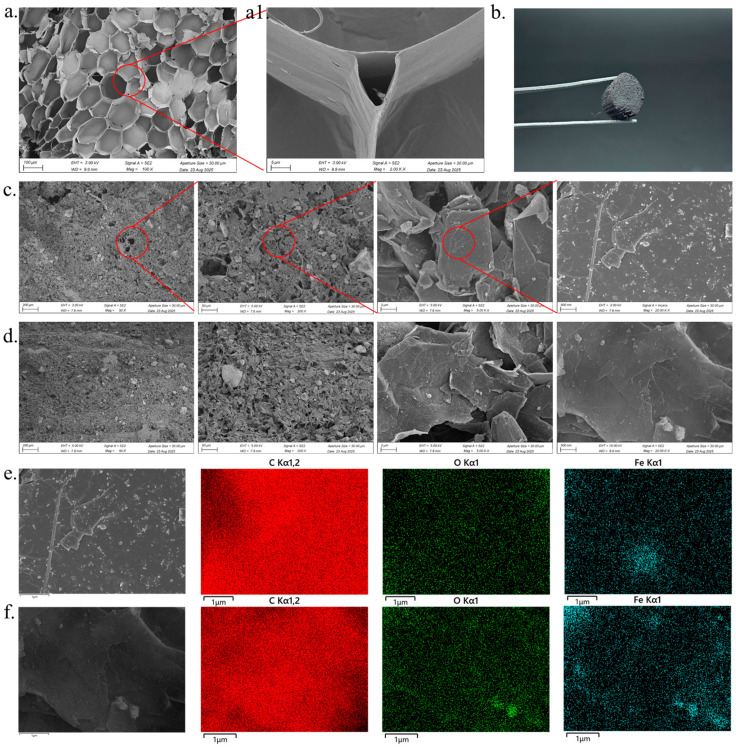
(**a**,**a1**) SEM images of CPDL; (**b**) digital photograph of Fe_3_O_4_/EG/PVDF-CPDL; (**c**) SEM images of the radial cross-section of Fe_3_O_4_/EG/PVDF-CPDL; (**d**) SEM images of the lateral surface of Fe_3_O_4_/EG/PVDF-CPDL; (**e**) EDS mapping of the radial cross-section of Fe_3_O_4_/EG/PVDF-CPDL; (**f**) EDS mapping of the lateral surface of Fe_3_O_4_/EG/PVDF-CPDL.

Following delignification treatment, the characteristic parenchyma tissue morphology of corn pith is preserved, while pore sizes are significantly enlarged and channel openness is markedly enhanced. Cross-sectional images of delignified corn pith (CPDL) (Figure 2a) reveal that lignin removal induces partial dissolution of cell wall components, resulting in wall thinning, a generalized increase in lumen dimensions of the original honeycomb-like parenchyma cells, and more clearly defined cellular boundaries. These uniformly distributed and interconnected lumens form a well-interconnected micron-scale porous network, providing ample spatial capacity and robust structural support for oil adsorption and storage. To quantitatively corroborate the morphological observations from SEM, mercury intrusion porosimetry (MIP) was employed to characterize the pore structure parameters. As shown in Figure S3a, the total pore volume of the delignified corn pith (CPDL) reached 39.37 mL/g, a significant four-fold increase compared to that of the raw corn pith (~9.62 mL/g). This substantial enhancement confirms that lignin removal not only eliminated the filling components within the cell walls but also successfully opened up the originally dense cellular network, thereby providing ample accommodation space for subsequent oil adsorption. Furthermore, differential analysis of the pore size distribution curves (derived from Figure S3a) reveals a distinct shift in pore hierarchy (Figure S3b). The raw corn pith exhibited a typical microporous structure, with pore sizes predominantly distributed in the 0.5–5 μm range. In contrast, the CPDL sample displayed a remarkable migration of the main pore distribution towards the macroporous regime, spanning 50–500 μm. This indicates that the chemical pretreatment did more than simply increase pore quantity; it fundamentally regulated the pore size hierarchy, transforming the material from a micropore-dominated structure to a macropore-dominated hierarchical architecture. Such a structural evolution allows oil to penetrate deeper into the pith interior through these enlarged channels with significantly reduced flow resistance, thereby markedly enhancing permeation efficiency. The connectivity between vascular bundle channels and adjacent cellular pores is effectively improved by lignin removal, thereby significantly enhancing the overall interconnectivity of the three-dimensional transport pathways within the material. This structural configuration facilitates rapid axial penetration of oils and their subsequent lateral diffusion through the pore network into surrounding parenchyma storage regions, achieving synergistic enhancement of adsorption and storage performance. Delignification treatment preserves the natural hierarchical interconnected architecture of corn pith while further enlarging pore dimensions and improving channel interconnectivity, thereby establishing a superior structural foundation for its application as a high-efficiency oil-absorbing material and offering a reliable structural basis for subsequent performance optimization.

The photographs of the samples (Figure 2b) demonstrate that the porous skeleton structure of the CPDL substrate is completely enveloped by a continuous and dense coating layer after the coating treatment, with no observable exposed substrate regions, thereby confirming the uniform deposition of the Fe_3_O_4_/EG/PVDF composite material on the CPDL surface. Scanning electron microscopy (SEM) observations of the Fe_3_O_4_/EG/PVDF-CPDL composite material (Figure 2c,d) reveal that the lamellar expanded graphite (EG) is tightly integrated with the polymer matrix, while the Fe_3_O_4_ nanoparticles are uniformly dispersed within the matrix, collectively forming a stable composite coating. In the preparation of the Fe_3_O_4_/EG composite photothermal conversion coating, polyvinylidene fluoride (PVDF) was incorporated as a binder to enhance the interfacial adhesion between the Fe_3_O_4_/EG composite coating and the delignified corn pith (CPDL) matrix. Compared with the control group without PVDF addition (Supplementary Figure S1), which exhibited uneven coating distribution, particle agglomeration, and severe delamination, the introduction of PVDF significantly improved the coating uniformity, density, and interfacial bonding to the porous substrate. This indicates that PVDF effectively acted as both a binding agent and a stabilizer, promoting homogeneous dispersion and anchoring of functional particles, while potentially preventing excessive infiltration of particles into deeper CPDL pores through its film-forming properties, thereby avoiding structural degradation caused by pore clogging. To verify the spatial distribution of functional components, elemental mapping via energy-dispersive X-ray spectroscopy (EDS) was further conducted. The C and Fe element distribution maps (Figure 2e,f) show a highly uniform dispersion of these elements across the observation area. Specifically, the homogeneous Fe signal confirms the absence of significant Fe_3_O_4_ nanoparticle agglomeration within the coating, while the widespread C distribution corresponds to contributions from the CPDL substrate, EG, and PVDF. The uniform element distribution corroborates the dense coating morphology observed via SEM, confirming the successful fabrication of a compositionally homogeneous Fe_3_O_4_/EG/PVDF composite photothermal layer. Furthermore, SEM images reveal that the composite coating forms hierarchical micro–nano-scale rough structures on the CPDL surface. This multi-scale roughness, comprising nano-sized Fe_3_O_4_ particles and micron-sized EG flakes embedded in the PVDF matrix, creates a light-trapping architecture. Such a structure significantly increases the effective surface area for photon incidence and, more importantly, induces multiple internal scattering of incident light within the coating layer. This scattering effect prolongs the optical path length, thereby enhancing light absorption across a broad spectrum (from UV to NIR), which is crucial for efficient solar-to-thermal conversion. This architecture establishes an essential morphological foundation for the material’s subsequent photothermal performance.

The SEM observations revealed that the deposited Fe_3_O_4_/EG/PVDF composite did not form a fully dense, pore-free film, but instead adhered to the CPDL skeleton as a porous overlayer. The coating layer itself exhibited micro–nano-scale rough structures with abundant fine cracks and interstices. These microscopic cracks and gaps interconnected with the inherent open pores of the CPDL substrate, together forming a three-dimensional transport network spanning the coating. This structural morphology can be analogized to the canopy layer of forest trees, where the micro-cracks on the coating surface resemble the “canopy shyness” phenomenon in nature. Specifically, the coating formed locally separated lamellar or island-like structures rather than a fully dense continuous membrane. This biomimetic architecture confers dual advantages: First, the composite coating with high specific surface area provides ample effective surface area for photothermal conversion; Second, the inherent porosity of the coating and the permeability of the substrate are preserved, which facilitates rapid fluid transport (e.g., oil adsorption/desorption). Such a hierarchical structure retaining macroscopic pores while incorporating a micro-rough photothermal layer establishes an ideal framework for achieving efficient photothermal-driven interfacial evaporation and oil–water separation. In summary, this composite coating architecture significantly enhances surface photothermal performance while maintaining the permeability and transport functions of the porous substrate, thereby constructing an optimal material platform for subsequent hydrophobic oil adsorption and photothermal evaporation applications.

### 3.2. Analysis of Structure and Composition of Materials

**Figure 3:**
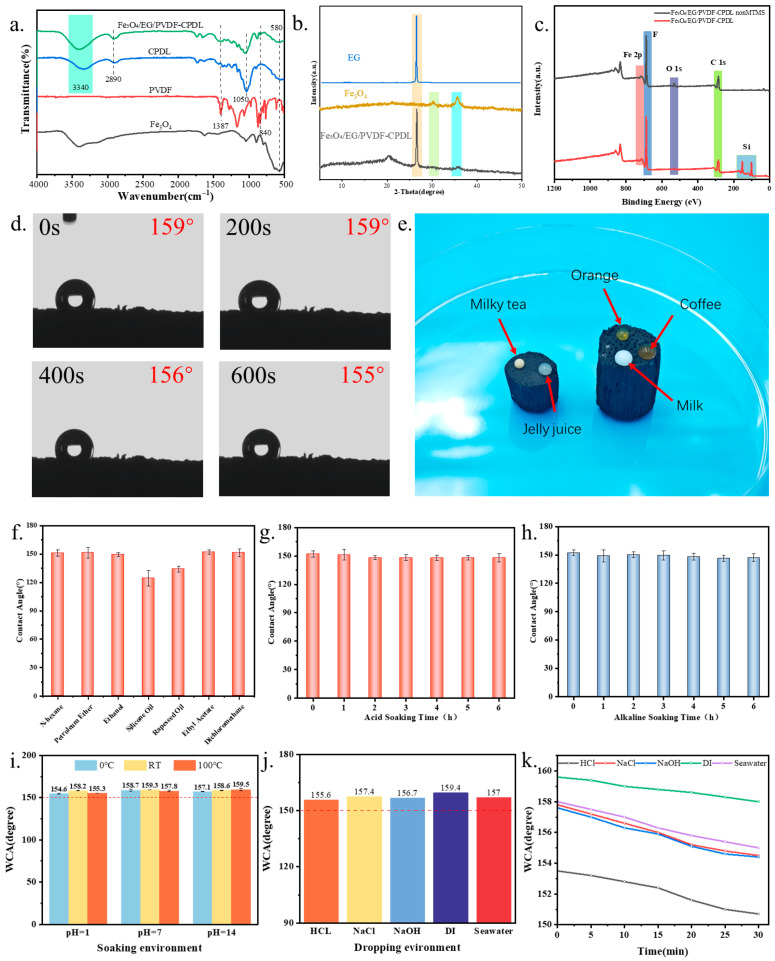
(**a**) FTIR spectra of CPDL, PVDF, Fe_3_O_4_, and Fe_3_O_4_/EG/PVDF-CPDL; (**b**) XRD patterns of EG, Fe_3_O_4_, and Fe_3_O_4_/EG/PVDF-CPDL; (**c**) XPS survey spectrum of Fe_3_O_4_/EG/PVDF-CPDL; (**d**) WCA of Fe_3_O_4_/EG/PVDF-CPDL; (**e**) wetting behavior of various liquid droplets on Fe_3_O_4_/EG/PVDF-CPDL; (**f**) contact angle of Fe_3_O_4_/EG/PVDF-CPDL after cyclic absorption of seven organic solvents/oils; (**g**) WCA of Fe_3_O_4_/EG/PVDF-CPDL after immersion in acid at pH = 1 for different times; (**h**) WCA of Fe_3_O_4_/EG/PVDF-CPDL after immersion in alkali at pH = 13 for different times; (**i**) WCA of Fe_3_O_4_/EG/PVDF-CPDL after treatment at different pH levels and temperatures; (**j**) WCA of Fe_3_O_4_/EG/PVDF-CPDL in different aqueous environments; (**k**) WCA of Fe_3_O_4_/EG/PVDF-CPDL at different times.

The FTIR spectra (Figure 3a) of pure CPDL exhibited characteristic absorption bands at 3340 cm^−1^ (O–H stretching vibration of cellulose), 2890 cm^−1^ (C–H stretching vibration), and 1050 cm^−1^ (C–O–C stretching vibration). For the Fe_3_O_4_/EG/PVDF-CPDL composite, additional peaks were observed: a distinct Fe–O vibration peak at 570 cm^−1^ confirmed Fe_3_O_4_ incorporation, while the absorption at 1580 cm^−1^ corresponded to aromatic sp^2^ C=C skeletal vibrations in EG. Characteristic PVDF absorption bands were also identified: peaks at 763 cm^−1^ and 795 cm^−1^ were attributed to the α-phase crystallinity, whereas those at 840 cm^−1^ and 1279 cm^−1^ corresponded to the β-phase crystallinity. The bands at 1169 cm^−1^ and 1387 cm^−1^ originated from CH_2_/CF_2_ vibrational modes in PVDF.

Beyond confirming the presence of individual components, the FTIR spectra provide insights into potential interfacial interactions within the composite. The broad O–H stretching band (~3340 cm^−1^) of cellulose in CPDL may experience subtle shifts or broadening in the composite spectrum, suggesting the formation of hydrogen bonding interactions between the hydroxyl groups on the CPDL surface and the fluorine atoms in PVDF (C–F⋯H–O) or the oxygen-containing groups on Fe_3_O_4_ nanoparticles. These interactions are crucial for achieving strong interfacial adhesion between the hydrophobic PVDF matrix and the hydrophilic biomass scaffold. Furthermore, the aromatic C=C vibrations from EG and the CH_2_/CF_2_ modes from PVDF likely engage in van der Waals and hydrophobic interactions, promoting the homogeneous dispersion of EG within the PVDF binder and enhancing the overall cohesion of the composite coating. Collectively, these interfacial forces—hydrogen bonding, van der Waals, and hydrophobic interactions—facilitate the stable integration of the functional (Fe_3_O_4_/EG/PVDF) coating onto the CPDL substrate, which is fundamental to the material’s structural integrity and durability.

XRD analysis further revealed the crystalline architecture of the composite (Figure 3b). The CPDL diffractogram displayed characteristic cellulose I-type diffraction peaks at 2θ = 16.2° and 22.3°, which remained present in the composite, demonstrating preservation of the cellulose crystalline framework after delignification and subsequent coating processes. New diffraction peaks emerged in the composite at 30.2°, 35.5°, 43.3°, 57.3°, and 62.9°, perfectly matching the (220), (311), (400), (511), and (440) planes of the Fe_3_O_4_ spinel structure (JCPDS No. 19-0629). The sharp and intense (311) peak indicated high crystallinity of the loaded Fe_3_O_4_. Notably, no distinct diffraction peaks for EG (~26.5°) or PVDF (~20.3°) were observed, likely due to their low loading content, poor crystallinity, or highly dispersed amorphous state within the composite matrix.

XPS analysis was performed to systematically compare the surface chemical composition before and after MTMS modification. Figure 3c presents the survey spectra of both Fe_3_O_4_/EG/PVDF-CPDL (non-MTMS) and Fe_3_O_4_/EG/PVDF-CPDL (with MTMS) samples. Both spectra exhibit dominant C 1s (~284.8 eV) and O 1s (~531 eV) signals originating from the CPDL substrate and surface oxygen-containing functional groups. A distinct F 1s peak (~688 eV) is observed in both samples, verifying the successful surface enrichment of PVDF from the composite coating. Additionally, Fe 2p signals (Fe 2p_3_/_2_ ~711 eV, Fe 2p_1_/_2_ ~724 eV) confirm the presence of Fe_3_O_4_ nanoparticles within the coating layer. Notably, the MTMS-modified sample displays two additional peaks at ~150 eV and ~101 eV that are absent in the non-MTMS spectrum, corresponding to Si 2s and Si 2p, respectively. The emergence of these Si signals provides direct evidence of successful MTMS vapor-phase deposition and the formation of a methyl-terminated siloxane network on the material surface. The coexistence of C, O, F, Fe, and Si elements in the XPS survey spectrum of the final product corroborates the FTIR and XRD findings, demonstrating the successful multi-step construction of the Fe_3_O_4_/EG/PVDF composite coating followed by MTMS surface functionalization on the CPDL scaffold.

### 3.3. Evaluation of Superhydrophobic Performance and Stability

#### 3.3.1. Superhydrophobic Performance and Its Universality

To validate the hydrophobic performance of Fe_3_O_4_/EG/PVDF-CPDL samples, water contact angle (WCA) measurements were performed. The Fe_3_O_4_/EG/PVDF-CPDL material exhibited excellent apparent superhydrophobicity, with a WCA of 155.3 ± 1.1° maintained even after 600 s of water droplet residence on its surface (Figure 3d). This superhydrophobicity showed broad universality for various aqueous liquids: water droplets, coffee, tea, and fruit juices all maintained near-spherical morphologies with distinct water-repellent behavior on the material surface (Figure 3e). Furthermore, a water absorption test indicated a rate of less than 1.5% after 24 h of immersion, confirming the material’s strong water repellency and structural integrity during prolonged water exposure.

To systematically elucidate the individual contributions of each fabrication step to the final superhydrophobic performance, water contact angle measurements were conducted on three representative samples corresponding to key modification stages: the pristine CPDL substrate, the Fe_3_O_4_/EG/PVDF-CPDL composite before MTMS modification (non-MTMS), and the final Fe_3_O_4_/EG/PVDF-CPDL product after MTMS vapor-phase deposition. As shown in Figure S5, the CPDL substrate exhibits pronounced hydrophilicity with an initial water contact angle of only 20.4°, and the water droplet completely penetrates into the porous structure within 0.8 s. This rapid wetting behavior is attributed to the abundant exposed cellulose hydroxyl groups on the fiber surface after delignification, which form strong hydrogen bonds with water molecules [31]. After coating with the Fe_3_O_4_/EG/PVDF composite slurry, the contact angle increases significantly to 121° and remains stable over 30 s, indicating a transition from hydrophilic to hydrophobic surface characteristics. This improvement is primarily ascribed to two synergistic effects: first, the inherent low surface energy of PVDF arising from its C-F bonds (surface energy ~25 mN/m) [32], and second, the micro/nano-scale roughness introduced by the Fe_3_O_4_/EG particles embedded in the PVDF matrix, which amplifies the hydrophobicity according to the Cassie–Baxter model [6,27]. However, this level of hydrophobicity is insufficient for effective oil–water separation applications. The subsequent MTMS vapor-phase deposition further elevates the contact angle to 159° with no observable change over 30 s, achieving robust superhydrophobicity. The methyl-terminated siloxane network formed on the surface reduces the surface energy to approximately 22 mN/m and stabilizes the air pockets trapped within the hierarchical porous structure [22,33]. These stepwise contact angle data conclusively demonstrate that while the PVDF-containing composite coating provides the foundational hydrophobicity and surface roughness, the MTMS modification is indispensable for achieving the superhydrophobic state required for high-efficiency oil–water separation [17,19]. The excellent temporal stability of the contact angles for both coated samples (no degradation over 30 s) further confirms the strong adhesion between the coating layers and the CPDL substrate, which is critical for maintaining performance during practical application cycles.

#### 3.3.2. Hydrophobic Retention After Cyclic Treatment in Different Organic Solvents/Oil Media

To evaluate the recyclability and hydrophobic stability of Fe_3_O_4_/EG/PVDF-CPDL in practical applications, this study further investigated its water contact angle (WCA) evolution after 10 cycles of adsorption–desorption with seven typical organic solvents/oils (n-hexane, petroleum ether, ethanol, silicone oil, rapeseed oil, ethyl acetate, and dichloromethane). The results (Figure 3f) demonstrated that the material maintained a stable WCA around 150° after repeated adsorption–desorption cycles with low-viscosity solvents (n-hexane, petroleum ether, ethanol, ethyl acetate, and dichloromethane), confirming its excellent superhydrophobic durability and structural robustness. For high-viscosity oils (silicone oil and rapeseed oil), the WCA decreased to approximately 130°, remaining within the hydrophobic range. This slight reduction was attributed to residual oil films adhering at fiber-coating interfaces, which increased surface free energy and weakened water repellency. Nevertheless, the 130° WCA still validated the material’s hydrophobic capability, and prolonged desorption time could partially restore the original contact angle. These findings indicate that the Fe_3_O_4_/EG/PVDF composite coating remains firmly anchored on the corn pith substrate during multiple cycles, preserving both micro/nano-roughened structures and low surface energy characteristics to effectively inhibit water penetration. In summary, the Fe_3_O_4_/EG/PVDF-CPDL composite exhibits stable hydrophobicity and recyclability for diverse oil types, particularly demonstrating exceptional superhydrophobicity endurance in low-viscosity solvent treatments, thereby providing critical experimental and theoretical support for practical engineering applications in complex aquatic environments.

#### 3.3.3. Chemical and Environmental Stability

To evaluate the material’s applicability in harsh environments, its chemical and environmental stability were systematically investigated. As shown in Figure 3g, after immersing Fe_3_O_4_/EG/PVDF-CPDL samples in acidic solution (pH = 1, HCl) for 2 h, the WCA slightly decreased from ~155° to 151°, yet remained within the superhydrophobic regime. Similar WCA stability was observed after alkaline solution immersion (pH = 14, NaOH). Throughout this initial testing period, no significant coating detachment, bubbling, cracking, or substrate exposure was observed, confirming the preservation of the macroscopic morphology. These results demonstrate that the MTMS vapor-phase deposition modified Fe_3_O_4_/EG/PVDF-CPDL maintains high hydrophobic performance in strong acids and alkalis, exhibiting excellent chemical durability.

This superior chemical stability arises from three synergistic mechanisms. First, MTMS molecules undergo hydrolytic condensation reactions on the composite coating surface and substrate micropores, forming a siloxane (Si-O-Si) network. The methyl end groups of this network impart extremely low surface free energy, effectively blocking water adsorption and spreading to reinforce superhydrophobicity. Second, as the binding medium, PVDF not only firmly anchors Fe_3_O_4_/EG composite particles onto the corn pith substrate, enhancing interfacial stability, but also provides additional corrosion resistance against acid/base attack due to its inherent chemical inertness. Third, although delignified corn pith retains an open porous structure, the complete Fe_3_O_4_/EG/PVDF coating forms an effective barrier between fibers and corrosive media, preventing capillary water uptake and interfacial delamination. Notably, the WCA under alkaline conditions (~148°, Figure 3h) was slightly lower than under acidic conditions, likely due to partial Si–O–Si bond cleavage in the MTMS monolayer or methoxy group hydrolysis under strong alkalinity. Nevertheless, the WCA remained above 148°, confirming sufficient alkali resistance for conventional oily wastewater treatment scenarios.

To further assess the material’s acid/base resistance and salt tolerance in real marine environments, comprehensive corrosive medium immersion tests were conducted. Aqueous solutions with pH = 1 (HCl), pH = 7 (deionized water), pH = 14 (NaOH), 3.5 wt% NaCl solution, and natural seawater were prepared. Sample specimens were completely immersed in 50 mL of each test solution in sealed glass containers at room temperature. For short-term stability assessment, samples were immersed for 15 min. For long-term stability assessment, samples were immersed for 24 h. After removal from the immersion solutions, samples were gently rinsed with deionized water, dried with nitrogen gas, and allowed to equilibrate at room temperature for 5 min before WCA measurement. As shown in Figure 3i, after 24-h immersion in different pH solutions (pH = 1, 7, 14), WCA measurements showed negligible changes (<3°), with all values remaining above 150°. Subsequent thermal cycling tests (0 °C, room temperature, 100 °C for 12 h each) confirmed WCA remained above 150°, demonstrating stable superhydrophobicity across wide temperature ranges and pH conditions. Further investigations into corrosive liquid resistance (HCl, NaCl, NaOH, and natural seawater) revealed minimal WCA variation (<3°) even after 15-min exposure (Figure 3j). Additionally, time-dependent WCA measurements were performed to evaluate the stability of water droplets on the material surface over extended periods. The same droplet was continuously observed and photographed at 60-s intervals for up to 30 min (Figure 3k). A slight decrease in WCA (<3°) was noted during extended droplet residence time. This phenomenon is primarily attributed to natural water droplet evaporation rather than water adsorption by the material, as evidenced by the maintained Cassie–Baxter wetting state with WCA above 150° throughout the observation, visible reduction in droplet volume consistent with evaporation, and the absence of water residue or penetration on the material surface post-test. According to the Young–Laplace equation, as droplet volume decreases due to evaporation, the contact angle can exhibit slight variations while the contact line remains pinned. This is a normal physical phenomenon and does not indicate degradation of superhydrophobic performance.

### 3.4. Oil Adsorption Performance Test

**Figure 4:**
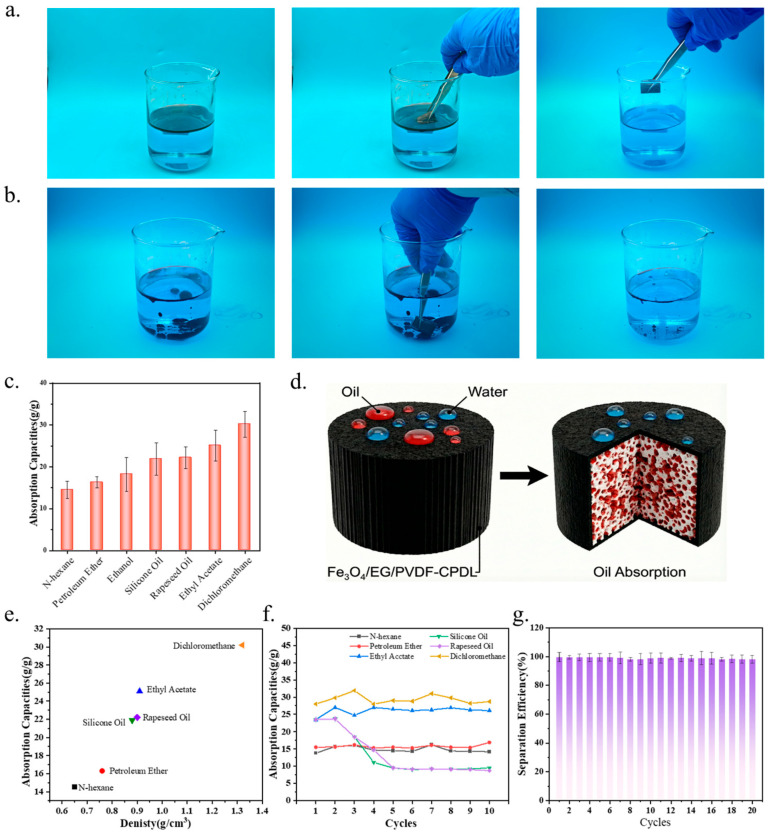
(**a**) Digital test of light oil–water separation; (**b**) Digital test of heavy oil–water separation; (**c**) Adsorption capacity of Fe_3_O_4_/EG/PVDF-CPDL for different organic solvents; (**d**) Schematic diagram of the oil–water separation mechanism; (**e**) Relationship between the adsorption capacity of Fe_3_O_4_/EG/PVDF-CPDL and the density of different organic reagents; (**f**) Cyclic oil adsorption capacity for six organic solvents/oils; (**g**) Cyclic separation efficiency of dichloromethane–water.

To systematically evaluate the lipophilic selectivity and oil adsorption capacity of Fe_3_O_4_/EG/PVDF-CPDL in aqueous environments, this study selected n-hexane (density water) as representative model pollutants for oil–water separation demonstrations and quantitative adsorption tests. The experimental procedures and results are illustrated in Figure 4a. For light oil adsorption, n-hexane and deionized water were sequentially added into a beaker at room temperature to establish a clear oil–water biphasic stratified system. Using tweezers, the Fe_3_O_4_/EG/PVDF-CPDL sample was brought into contact with the oil–water interface. Benefiting from the material’s superhydrophobicity and strong oleophilicity, the oil phase was rapidly absorbed into the porous structure upon contact, completing the adsorption process within seconds. Post-adsorption, the water phase remained clear without visible oil films or residues, confirming the material’s high-efficiency light oil adsorption capability. For heavy oil adsorption, dichloromethane naturally settled at the bottom of the beaker, forming a distinct oil layer. The Fe_3_O_4_/EG/PVDF-CPDL sample was immersed into the water phase and guided to the oil layer at the bottom. Upon contact, the material exhibited rapid adsorption response. After removal, nearly complete oil phase elimination was observed, with water clarity maintained (Figure 4b). These results demonstrate that Fe_3_O_4_/EG/PVDF-CPDL possesses strong affinity and fast adsorption kinetics for oils of varying densities, fully exhibiting selective lipophilic characteristics. Based on its superhydrophobic/underwater superoleophobic properties, the separation mechanism can be explained as follows: when water droplets contact the Fe_3_O_4_/EG/PVDF-CPDL surface, the micro/nano-structured roughness forms an air layer that effectively isolates water molecules, preventing their penetration into the material and significantly reducing actual water–material contact area. Oils, with low surface tension, rapidly wet and permeate the internal porous network, enabling efficient oil–water separation (Figure 4d).

To quantitatively characterize adsorption performance, seven organic liquids with diverse physicochemical properties (polarity, viscosity, density) were tested: n-hexane, petroleum ether, ethanol, silicone oil, rapeseed oil, ethyl acetate, and dichloromethane. The saturated adsorption capacities are summarized in Figure 4c,d. The material exhibited excellent adsorptions for all tested liquids, with capacities ranging from 14.5 to 30.2 g/g. Combined with the ~155° water contact angle, these results confirm the material’s broad-spectrum and high-efficiency adsorption potential while maintaining extreme hydrophobicity. Further analysis revealed several key correlations: 1. Density Effect: Denser organic solvents (e.g., dichloromethane, ρ = 1.33 g/cm^3^) exhibited significantly higher gravimetric capacities (30.2 g/g) compared to lighter solvents (e.g., n-hexane, ρ = 0.66 g/cm^3^; 14.5 g/g). This aligns with porous material adsorption principles: under fixed pore volume, higher oil density translates to greater mass adsorption. The gravimetric adsorption capacity (Q) can be expressed as Q = V_pore_ × ρ_oil_, where V_pore_ represents the effective pore volume accessible to the adsorbate and ρ_oil_ is the liquid density. 2. Viscosity Effect on Recyclability: While viscosity showed limited influence on initial saturated adsorption capacity, it significantly affected cyclic stability. For low-viscosity, volatile solvents (n-hexane, petroleum ether, ethyl acetate), the material maintained stable saturated capacities over 10 cycles. However, for viscous oils (silicone oil, rapeseed oil), gradual capacity decline occurred during multiple cycles. This was attributed to residual viscous oil in pores: poor fluidity hindered complete removal via volatilization, gravity drainage, or ethanol washing, with residual films progressively occupying pore spaces and reducing effective adsorption sites. 3. Polarity Independence: The material showed comparable adsorption capacities for both polar (ethanol, dielectric constant ε = 24.5; ethyl acetate, ε = 6.0) and non-polar (n-hexane, ε = 1.9; dichloromethane, ε = 9.1) organic liquids. This indicates that the adsorption mechanism is predominantly governed by capillary forces and van der Waals interactions rather than specific polar interactions. The hydrophobic PVDF/EG surface minimizes competitive water adsorption while allowing unrestricted uptake of organic liquids regardless of their polarity, which is advantageous for practical oil spill remediation where oil composition varies widely.

To further elucidate the individual contributions of each functionalization step to adsorption capacity, this study systematically compared the saturated adsorption capacities of three representative oils (n-hexane, rapeseed oil, and dichloromethane) across three samples at different modification stages: pristine CPDL, Fe_3_O_4_/EG/PVDF-CPDL without MTMS silanization (non-MTMS), and the final MTMS-modified Fe_3_O_4_/EG/PVDF-CPDL product (Figure S3). The results show that pristine CPDL exhibited the highest adsorption capacity for all tested oils, primarily attributed to its maximum inherent pore volume unoccupied by any functional coating. After introducing the Fe_3_O_4_/EG/PVDF functional coating (non-MTMS group), adsorption capacities for all oils decreased by approximately 13–18%, which is mainly attributed to two physical mechanisms: (1) the coating material adheres to the cellulose skeleton surface and pore channel walls, occupying partial effective pore volume and directly reducing the pore capacity available for oil filling, and (2) the coating increases pore channel transport resistance, affecting oil permeation rate and filling completeness within the pore network. This quantitatively demonstrates the “pore volume cost” paid for integrating photothermal and magnetic functionalities. Subsequent MTMS vapor-phase deposition hydrophobization treatment resulted in a slight capacity recovery of approximately 4–6%. This recovery effect is attributed to the formation of ultra-low surface energy methyl (-CH_3_) terminals on the material surface (including pore inner walls) through MTMS modification. Additionally, MTMS deposits as vapor-phase molecules, forming a monomolecular-level chemical modification layer that does not significantly alter the material’s pore size distribution and three-dimensional connectivity, rendering physical occupation effects negligible. This greatly optimizes oil wettability and ensures efficient, complete filling of remaining pores through enhanced capillary driving forces. Overall, the final product exhibits a net capacity decrease of approximately 10–15% relative to pristine CPDL. However, this trade-off is necessary and reasonable: while pristine CPDL has slightly higher capacity, it is hydrophilic and cannot achieve oil–water selective separation, nor does it possess magnetic recovery or photothermal assistance capabilities. In contrast, the functionally modified composite, while maintaining relatively high adsorption capacity, acquires superhydrophobic/superoleophilic selectivity, magnetically controlled recovery capability, and photothermal response functionality, making its comprehensive performance far more suitable for practical oil spill remediation scenarios.

To evaluate recyclability and long-term stability, repeated adsorption–desorption cycles were conducted. As shown in Figure 4f, for low-viscosity, volatile solvents (n-hexane, petroleum ether, ethyl acetate), the material maintained stable saturated capacities over 10 cycles, demonstrating excellent cyclic adsorption stability. However, for viscous oils (silicone oil, rapeseed oil), gradual capacity decline occurred during multiple cycles. This was attributed to residual viscous oil in pores: poor fluidity hindered complete removal via volatilization, gravity drainage, or ethanol washing, with residual films progressively occupying pore spaces and reducing effective adsorption sites. Furthermore, to investigate long-term operational stability, a 20-cycle oil–water separation test was performed using n-hexane-water mixtures. As shown in Figure 4g, after 20 continuous cycles, the oil–water separation efficiency remained above 96% without significant decay. No coating detachment or pore blockage was observed, confirming structural and functional integrity. In summary, Fe_3_O_4_/EG/PVDF-CPDL demonstrates not only broad-spectrum high-capacity adsorption for diverse oils but also exceptional cyclic stability and long-term reliability, establishing a solid experimental foundation for practical applications in complex oil spill response and water pollution remediation.

### 3.5. Photothermal Performance Test

**Figure 5:**
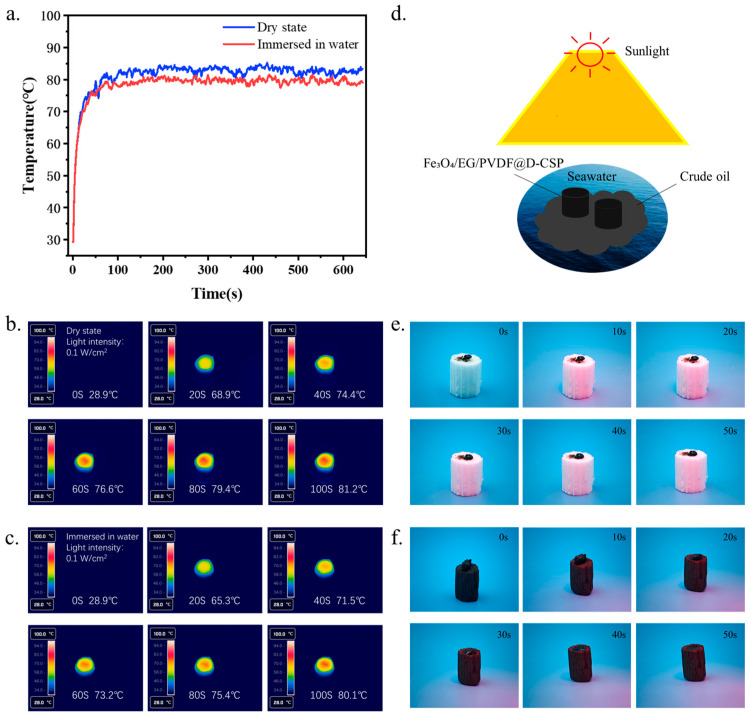
(**a**) Temperature variation curves of Fe_3_O_4_/EG/PVDF-CPDL under different states with light irradiation time; (**b**) Thermal infrared images of Fe_3_O_4_/EG/PVDF-CPDL in dry state showing temperature variation over light irradiation time; (**c**) Thermal infrared images of Fe_3_O_4_/EG/PVDF-CPDL in immersed state showing temperature variation over light irradiation time; (**d**) Schematic diagram of crude oil adsorption by Fe_3_O_4_/EG/PVDF-CPDL; (**e**) Digital photographs of crude oil adsorption by CPDL under simulated light source; (**f**) Digital photographs of crude oil adsorption by Fe_3_O_4_/EG/PVDF-CPDL under simulated light source.

To systematically evaluate the photothermal conversion performance of Fe_3_O_4_/EG/PVDF-CPDL and its potential application in heavy oil viscosity reduction, this study conducted tests under simulated solar illumination using a xenon lamp with a power density of 0.1 W·cm^−2^ (equivalent to one standard sun intensity). Temperature evolution of the sample surface was monitored under both dry conditions and partially submerged conditions. As shown in Figure 5a, the sample exhibited exceptional photothermal response from an initial ambient temperature of 28 °C. Under dry conditions, the Fe_3_O_4_/EG/PVDF-CPDL sample reached a surface temperature of 81.2 °C within 100 s of continuous illumination. The heating kinetics displayed a typical profile: the fastest temperature rise occurred in the first 50 s, followed by a gradual deceleration due to enhanced convective and radiative heat loss to the environment, ultimately reaching photothermal equilibrium at ~100 s. This rapid temperature increase confirms the composite coating’s efficient light-to-heat conversion capability. To simulate practical oil–water separation scenarios involving floating oil removal or interfacial evaporation, the photothermal performance was further tested with the sample edge immersed in water. Under submerged conditions, the surface temperature still reached 80.1 °C under identical illumination, closely matching the dry-state performance. This indicates that the presence of water had no significant inhibitory effect on the coating’s optical absorption and heat generation, primarily attributed to the material’s intrinsic superhydrophobicity, which limits actual water contact area, and the fact that photothermal conversion occurs predominantly within the coating interior, ensuring efficient thermal output even under aqueous conditions.

The exceptional photothermal performance (81.2 °C within 100 s) indirectly corroborates the material’s superior light absorption capability. According to the energy balance principle, such rapid temperature elevation under one-sun illumination (1000 W/m^2^) requires an average solar absorptance exceeding 90%, assuming typical convective and radiative heat loss coefficients. This high absorption efficiency is consistent with the hierarchical micro–nano rough structure observed in Section 3.1, where the lamellar EG and Fe_3_O_4_ nanoparticles create a ‘light-trapping’ architecture that minimizes reflectance through multiple internal scattering events. The black appearance of the composite coating (Figure 2b) further visually confirms its broadband light absorption characteristics, which is a well-documented feature of carbon-based photothermal materials. To further evaluate the long-term operational reliability of the photothermal function, the stability of the composite under repeated light irradiation cycles was investigated. The Fe_3_O_4_/EG/PVDF-CPDL sample was subjected to 10 consecutive on/off irradiation cycles (100 s illumination followed by natural cooling to ambient temperature per cycle) under one-sun intensity. As shown in Figure S4, the material demonstrated remarkable photothermal stability. The peak surface temperature in each cycle consistently reached above 80 °C, with no observable decay in the heating rate or maximum attainable temperature throughout the 10 cycles. This excellent cyclic stability confirms that the functional coating is resistant to potential photothermal degradation, oxidation, or delamination under repeated solar exposure, which is crucial for ensuring the material’s durability and practical viability in long-term, cyclic outdoor applications such as repeated oil spill remediation operations.

Dynamic temperature field distributions captured by infrared thermography (Figure 5b,c) further revealed the sample’s photothermal behavior. The images showed no observable temperature gradients across the surface during illumination, with no localized “hotspots” or “cold regions” caused by coating inhomogeneity or component agglomeration. This uniform temperature distribution directly corroborates the previous SEM and EDS analyses, confirming the formation of a continuous, homogeneous functional coating on the CPDL substrate through PVDF-mediated dispersion of Fe_3_O_4_ and EG. The superior photothermal performance of Fe_3_O_4_/EG/PVDF-CPDL arises from synergistic effects among its components and optimized structural design, where expanded graphite acts as a broadband absorber and high-efficiency thermal conductor with strong UV-Vis-NIR absorption and rapid heat diffusion through its in-plane high thermal conductivity, while Fe_3_O_4_ nanoparticles demonstrate enhanced visible-NIR light absorption and photothermal conversion through non-radiative processes (e.g., photo-induced electron/lattice relaxation) and promote multiple scattering to extend optical path length for improved light capture. PVDF serves as a critical polymer binder that uniformly disperses and firmly anchors Fe_3_O_4_/EG nanoparticles on the porous CPDL substrate, preventing agglomeration-induced photothermal quenching and ensuring mechanical stability, while its inherent chemical inertness guarantees long-term durability of the photothermal layer under harsh aqueous environments and prolonged irradiation.

In summary, Fe_3_O_4_/EG/PVDF-CPDL integrates the 3D porous adsorption architecture of the CPDL biomass substrate, robust superhydrophobicity conferred by MTMS/PVDF modification, and efficient, uniform photothermal conversion from the Fe_3_O_4_/EG/PVDF coating. This “adsorption–photothermal” integrated design enables not only physical oil adsorption but also in situ viscosity reduction in adsorbed heavy oils or enhanced desorption through photothermal heating, potentially driving interfacial water evaporation, thereby demonstrating a promising biomass-based solution for high-efficiency, active, and renewable oil spill remediation and oil–water separation technologies.

### 3.6. Mechanical Properties Test

**Figure 6:**
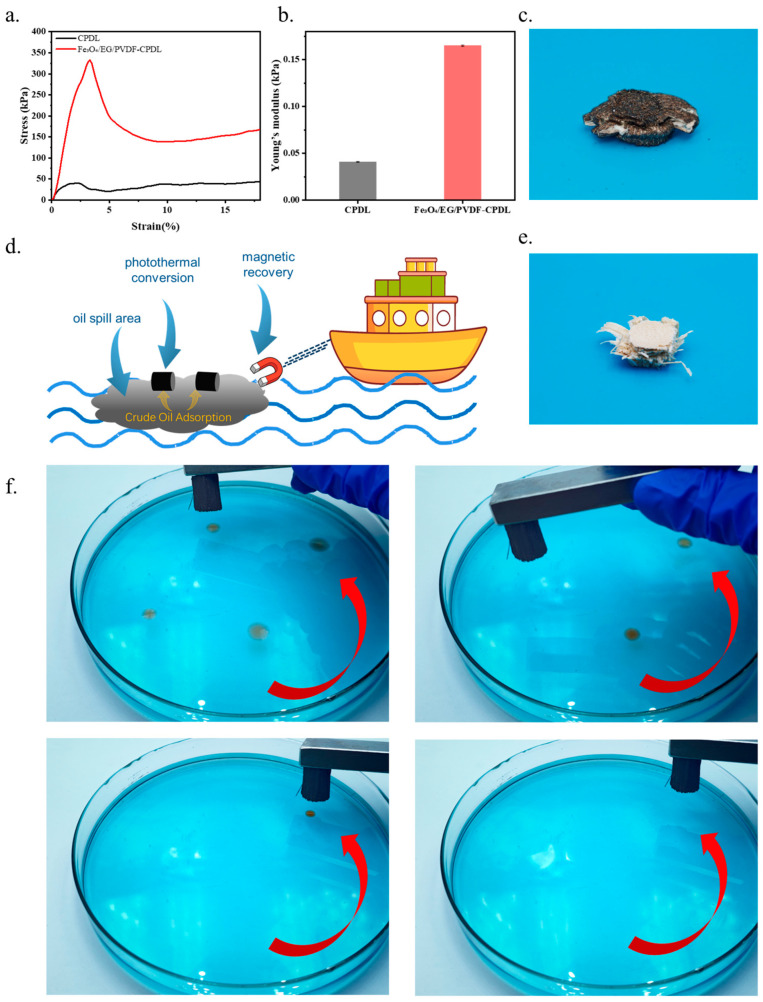
(**a**) Stress–strain curves of the samples; (**b**) Young’s modulus of the samples; (**c**) Digital photograph of Fe_3_O_4_/EG/PVDF-CPDL after compression test; (**d**) Schematic diagram of magnetic-driven crude oil adsorption and recovery; (**e**) Digital photograph of CPDL after compression test; (**f**) Fe_3_O_4_/EG/PVDF-CPDL adsorbing n-hexane in the direction of the arrow.

To systematically evaluate the structural stability and mechanical reliability of Fe_3_O_4_/EG/PVDF-CPDL in practical applications, this study conducted quasi-static compression tests on both the delignified corn pith substrate (CPDL) and the Fe_3_O_4_/EG/PVDF-CPDL sample after composite coating assembly. As shown in Figure 6a, the stress–strain curve of CPDL exhibited the classical three-stage characteristics of porous biomaterials: an initial elastic regime was followed by a prolonged plateau region corresponding to elastic buckling and progressive collapse of pore walls, and finally a densification stage with rapid stress escalation. In contrast, the stress–strain curve of Fe_3_O_4_/EG/PVDF-CPDL shifted toward higher stress levels, demonstrating significantly enhanced load-bearing capacity. The initial slope of the elastic regime increased markedly, indicating that the composite coating substantially improved the overall stiffness. During the plateau region, the reduced stress fluctuations confirmed strong interfacial adhesion between the coating and substrate, enabling collaborative load-bearing and preventing localized structural instability. The compressive strength of CPDL was 40 kPa, while Fe_3_O_4_/EG/PVDF-CPDL reached 320 kPa—eight times higher. Young’s modulus comparison was equally pronounced (Figure 6b): CPDL exhibited low modulus due to its loose porous structure, whereas Fe_3_O_4_/EG/PVDF-CPDL showed significantly enhanced modulus, reflecting improved resistance to deformation. The post-compression macroscopic morphology (Figure 6c,e) revealed distinct failure modes between the two materials. CPDL displayed uniform collapse, while Fe_3_O_4_/EG/PVDF-CPDL exhibited a unique staged failure mechanism: the outer Fe_3_O_4_/EG/PVDF composite coating first underwent regular folding buckling under the indenter, forming petal-like wrinkled patterns. As compression progressed, the internal CPDL substrate gradually flattened until full densification. This phenomenon illustrated the synergistic deformation mechanism of the coating–substrate composite structure—the rigid coating initially dissipated energy through buckling while uniformly transferring load to the porous interior, preventing stress-concentration-induced brittle fracture. The buckled petal-like wrinkles not only extended the deformation path but also prolonged the densification, thereby enhancing the overall energy absorption capacity.

The remarkable mechanical improvements primarily originated from the reinforcing effects of the composite coating and strong interfacial bonding. First, the PVDF binder not only firmly adhered Fe_3_O_4_ and EG particles but also infiltrated into the surface pores of CPDL, forming robust physical anchoring points after curing to ensure strong coating–substrate adhesion. Second, the dispersed Fe_3_O_4_ nanoparticles and expanded graphite platelets in the coating acted as rigid reinforcing phases that collectively bore external loads, directly contributing to the strength enhancement. This surface-dominated reinforcement strategy significantly improved stiffness and strength without blocking the main pores of CPDL, preserving its lightweight characteristics and adsorption functionality derived from the porous architecture.

Through constructing robust coating–substrate interfaces and introducing rigid reinforcing phases, Fe_3_O_4_/EG/PVDF-CPDL achieved substantial improvements in compressive strength and Young’s modulus while maintaining the lightweight porous nature of the substrate. Its unique out-to-in, buckling-dominated synergistic deformation mechanism endowed the material with excellent structural compliance and energy dissipation capability. This provides essential mechanical assurance for practical operations involving gripping, transportation, stacking, and recovery during oil pollution remediation processes.

To objectively evaluate the comprehensive performance of the as-prepared Fe_3_O_4_/EG/PVDF-CPDL composite in this work, a comparison of key performance parameters with several recently reported biomass-based photothermal oil-absorbing materials was conducted (as summarized in Table 1). Under the unified standard illumination condition (1 sun, 1000 W/m^2^), the as-prepared material exhibits excellent photothermal conversion efficiency with an equilibrium temperature up to 80.1 °C, which is higher than that of other similar materials listed in the table. This superior photothermal performance ensures its strong active viscosity reduction capability for high-viscosity crude oil. In terms of adsorption performance, the material achieves an ultrahigh adsorption capacity of up to 14.9 g/g for the model oil n-hexane, which is significantly superior to most of the compared materials. This result verifies the effectiveness of the three-dimensional porous structure constructed via delignification and coating optimization for oil storage. More importantly, in terms of integrated functionality, the as-prepared material successfully integrates three core functions into one system: efficient photothermal conversion, high adsorption capacity, and magnetically responsive recovery. In contrast, some materials listed in the table exhibit photothermal property or magnetic responsiveness but have obvious shortcomings in adsorption capacity, while others possess a certain adsorption capacity but lack photothermal conversion capability or magnetically responsive function. In summary, via elaborate material design, this work achieves the optimization and synergy of multiple properties while preserving the green characteristics of the biomass substrate. The resultant Fe_3_O_4_/EG/PVDF-CPDL composite exhibits stronger comprehensive application potential in the full process of “photothermal viscosity reduction–rapid adsorption–magnetically controlled recovery” for the remediation of high-viscosity floating oil pollution.

### 3.7. Magnetic Response Performance and Magnetic-Driven Recovery

Fe_3_O_4_/EG/PVDF-CPDL composite material exhibits significant magnetic responsiveness due to the incorporation of Fe_3_O_4_ nanoparticles, providing critical support for the controllable recovery of adsorbed materials after oil adsorption. To validate its magnetic actuation performance and practical potential in oil–water separation, simulated magnetic recovery and dynamic adsorption experiments were conducted.

First, a simulated scenario demonstrated the material’s magnetic recovery capability (Figure 6d). When placed near a simulated oil-contaminated water surface, the material could be directionally attracted toward the target zone upon activation of an underlying permanent magnet device. Under magnetic influence, the material not only achieved directional movement but also performed in situ adsorption upon reaching the oil-contaminated area, leveraging its superhydrophobic and oleophilic properties. After adsorption saturation, the oil-loaded material could be continuously retrieved from the surface via magnetic guidance, enabling integrated operation of directional movement, in situ adsorption, and final recovery without complex mechanical retrieval. This process highlights its potential for simplifying recovery procedures.

Further, the dynamic adsorption process of the material under magnetic actuation was demonstrated (Figure 6f). In a biphasic system comprising methylene blue-stained aqueous phase and Sudan III-stained n-hexane (dyes used solely for tracing, without altering oil–water phase separation or adsorption behavior), the Fe_3_O_4_/EG/PVDF-CPDL material could be precisely guided along a predefined counterclockwise path by moving an external magnet. Upon reaching the oil phase, it rapidly and completely adsorbed n-hexane. The oil-loaded material could then be continuously magnetically pulled for full recovery, including the adsorbed oil. This experiment directly confirmed the material’s excellent magnetic actuation capability and its ability to perform rapid adsorption–recovery during motion.

Collectively, these results demonstrate that the Fe_3_O_4_/EG/PVDF-CPDL composite, by integrating porous adsorption characteristics with magnetic responsiveness, offers a feasible solution for the “adsorption–recovery” closed-loop in oil spill remediation. Compared to traditional recovery methods relying on buoyancy or mechanical retrieval, this magnetic recovery strategy features precise control, strong directionality, and minimal structural disturbance to the material. It effectively promotes rapid aggregation and removal of adsorbed materials, reducing secondary dispersion after use, lowering environmental residue risks, and enhancing the efficiency and controllability of the entire oil spill response process. When combined with the material’s inherent photothermal and adsorption properties, this magnetic functionality further elevates its comprehensive application value in complex marine oil spills, particularly in scenarios requiring rapid response and efficient recovery.

## 4. Conclusions

This study successfully developed a multifunctional biomass-based oil-adsorbing material (Fe_3_O_4_/EG/PVDF-CPDL) using delignified corn pith (CPDL) as a three-dimensional porous substrate. The material was constructed through the integration of a Fe_3_O_4_/EG/PVDF composite functional coating and trimethoxysilane surface modification. The success of this design is primarily reflected in its stable composite architecture: Delignification preserved and optimized the natural interconnected porous structure of CPDL while providing an ideal scaffold for functional coatings. The Fe_3_O_4_/EG coating, bound by PVDF, was uniformly and robustly anchored to form a unique micro/nano-roughened surface structure. This architecture ensured efficient dispersion of photothermal components and strong interfacial adhesion while avoiding pore blockage. After modification, the material exhibited excellent superhydrophobicity (water contact angle of 155°), which remained stable across diverse media, multiple adsorption–desorption cycles, and simulated marine acid/base/salt corrosion environments. In terms of oil–water separation performance, the material demonstrated rapid and selective adsorption capabilities for both light and heavy oils, with saturation adsorption capacities ranging from 14.8 to 30.2 g/g for various oils. Remarkably, it maintained separation efficiency above 96% after 20 cycles. Synergistic interactions between Fe_3_O_4_ and expanded graphite endowed the material with significant photothermal conversion efficiency, enabling its surface temperature to exceed 80 °C within 100 s under one-sun illumination. This property achieved in situ viscosity reduction in high-viscosity crude oil (>95% viscosity reduction), allowing complete adsorption within 50 s—a critical advantage for heavy oil remediation. Mechanically, the composite coating enhanced compressive strength to eight times that of the pristine CPDL substrate. The buckling-dominated deformation mechanism ensured structural integrity during practical handling, stacking, and recovery operations. Moreover, the magnetic responsiveness imparted by Fe_3_O_4_ components enabled precise directional actuation and efficient recovery via external magnetic fields, offering a key solution for controlled post-adsorption collection and secondary pollution prevention. In summary, Fe_3_O_4_/EG/PVDF-CPDL integrates porous adsorption, superhydrophobic corrosion resistance, photothermal viscosity reduction, mechanical reinforcement, and magnetic-controlled recovery into a single platform. The synergistic interplay of these properties positions it as a promising candidate for high-efficiency, controllable oil spill remediation in complex marine environments. Future work should further validate its stability and closed-loop recovery reliability under realistic oceanic conditions, including wave action, oil emulsification, and long-term operational scenarios.

## Figures and Tables

**Table 1:** Performance comparison of the as-prepared material with previously reported biomass-based photothermal oil/water separation materials.

Material Description	Photothermal Performance (1 Sun)	Adsorption Capacity (g/g) (N-Hexane)	Magnetic Property	Reference
Sugarcane residue/polydopamine/fluorine-containing	76.3 °C	11.5	No	[34]
Carbonized yam/PDMS	78.2 °C	0.9	No	[35]
Sisal fiber cellulose/Ag_2_O@Ag/PDMS	58.8 °C (0.3 sun)	12	No	[36]
Wood sponge/Fe_3_O_4_/Polydopamine/PDMS	63.4	12.4	Yes	[37]
Lignin-based polyurethane/Fe_3_O_4_/OTMS	66.5	4	Yes	[38]
Corn stalk/Fe_3_O_4_/EG/PVDF/MTMS	80.1	14.9	Yes	This work

## Data Availability

The original contributions presented in this study are included in the article/Supplementary Materials. Further inquiries can be directed to the corresponding author.

## Supplementary Materials

The following supporting information can be downloaded at: https://www.mdpi.com/article/10.3390/polym18070860/s1, Figure S1: Digital photograph of the sample without PVDF coating; Figure S2: Variation trend of petroleum viscosity with temperature; Figure S3: (a) The cumulative intrusion amount of mercury in the mercury intrusion porosimetry test. (b) The incremental intrusion amount of mercury in the mercury intrusion porosimetry test; Figure S4: Maximum surface temperature of the sample during 10 consecutive on/off cycles of simulated solar irradiation; Figure S5: Static water contact angles of (a) CPDL, (b) Fe_3_O_4_/EG/PVDF-CPDL (non-MTMS), and (c) Fe_3_O_4_/EG/PVDF-CPDL (with MTMS).

## Abbreviations

The following abbreviations are used in this manuscript:

CPDL	delignified corn pith
PVDF	polyvinylidene fluoride
EG	expanded graphite
