# Supplementary material for: Fabrication of Magnetically and Photothermally Functionalized Materials Based on Corn Stalk Pith Framework for Oil–Water Separation
Source: Polymers (Basel). 2026 Mar 31;18(7):860. doi: 10.3390/polym18070860 (PMC13075026; doi:10.3390/polym18070860)
Supplement: Supplementary file 1 [file polymers-18-00860-s001.zip › polymers-4216503-Supplementary.pdf]

Supplementary Materials:

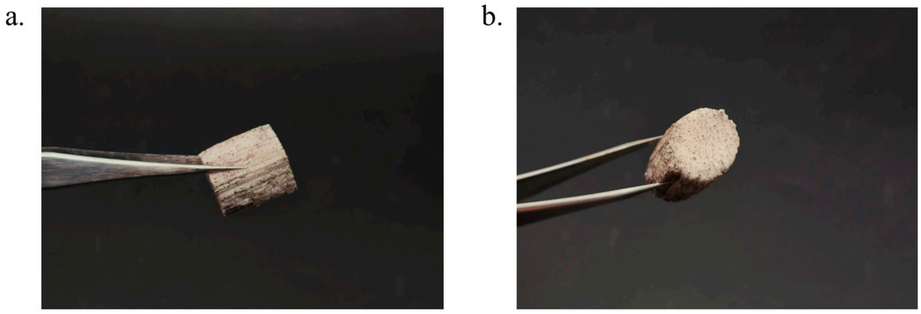

Figure S1: Digital photograph of the sample without PVDF coating

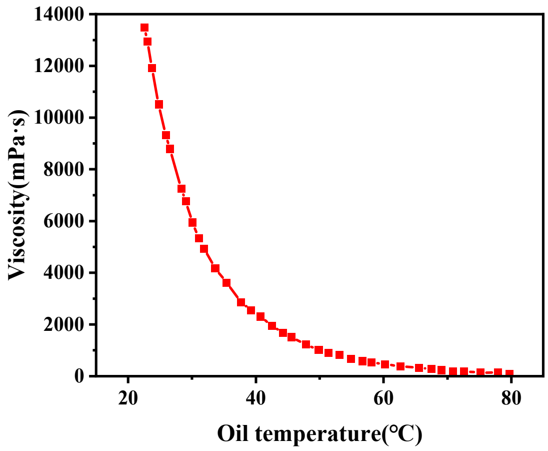

Figure S2: Variation trend of petroleum viscosity with temperature

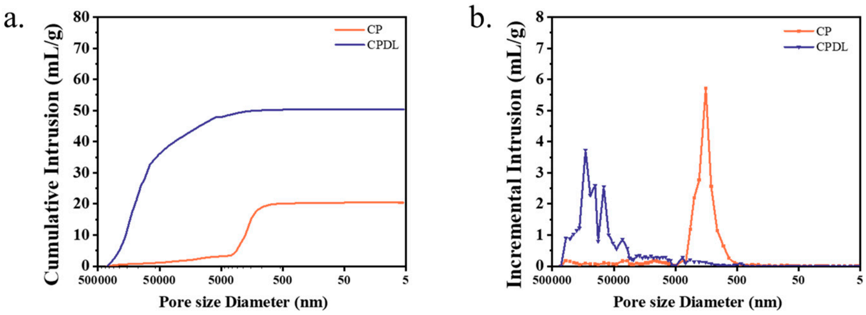

Figure S3: (a) The cumulative intrusion amount of mercury in the mercury intrusion porosimetry test. (b) The incremental intrusion amount of mercury in the mercury intrusion porosimetry test.

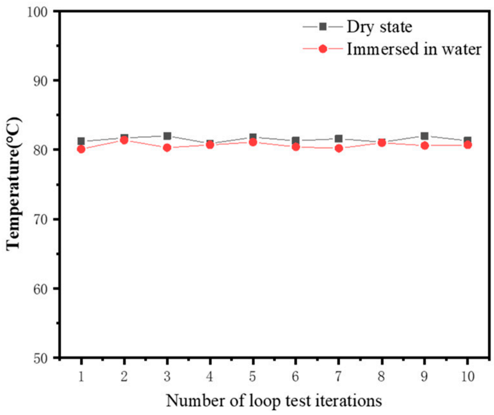

Figure S4. Maximum surface temperature of the sample during 10 consecutive on/off cycles of simulated solar irradiation.

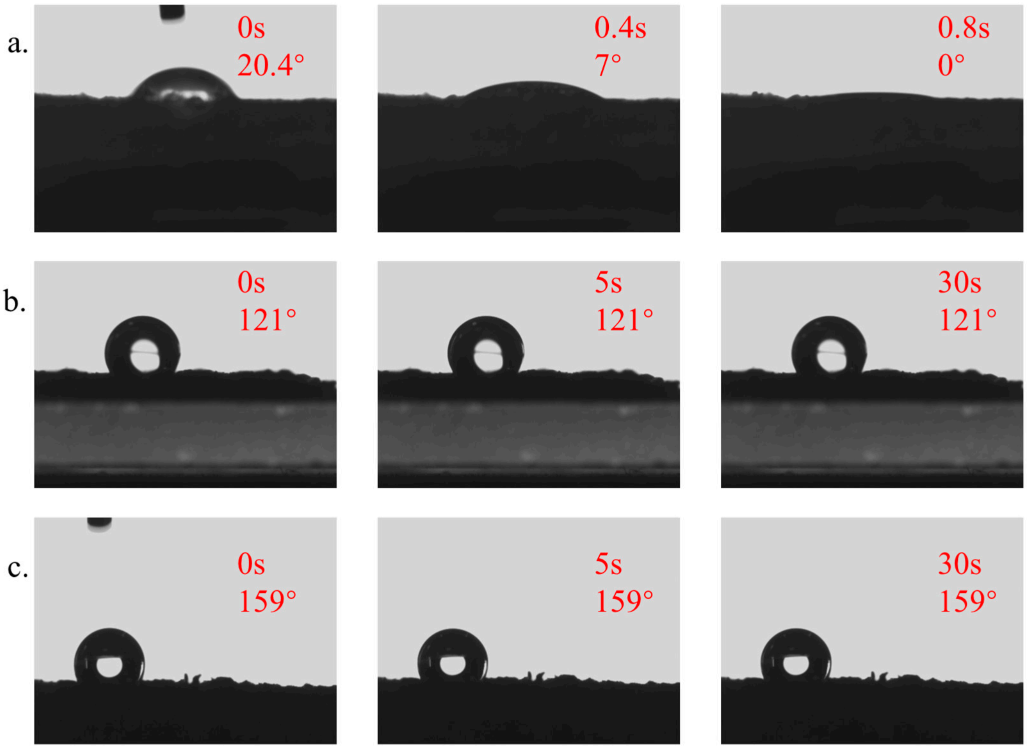

Figure S5. Static water contact angles of (a) CPDL, (b) Fe_3O_4/EG/PVDF-CPDL (non-MTMS),

and (c) Fe_3O_4/EG/PVDF-CPDL (with MTMS).
